# West Nile Virus and Other Nationally Notifiable Arboviral Diseases — United States, 2024

**DOI:** 10.15585/mmwr.mm7529a1

**Published:** 2026-07-30

**Authors:** Kimberly E. Mace, Daniel Jacobs, Carolyn V. Gould, J. Erin Staples, Shelby L. Lyons

**Affiliations:** ^1^Division of Vector-Borne Diseases, National Center for Emerging and Zoonotic Infectious Diseases, CDC; ^2^Alaka`ina Foundation, Orlando, Florida.

**Summary**

**What is already known about this topic?**

West Nile virus (WNV) is the leading cause of arthropodborne viral (arboviral) disease, manifesting as acute febrile or neurologic illness in the United States. Several other arboviruses transmitted through bites of infected mosquitoes or ticks also occur in the United States and cause similar illnesses. Transmission through blood transfusion or organ transplantation has been reported.

**What is added by this report?**

In 2024, arboviral disease cases were reported from 48 states and the District of Columbia. WNV was most common, followed by Powassan virus disease, which increased to a new record high. La Crosse virus disease was the most common arboviral disease among children; the case-fatality rate was highest for eastern equine encephalitis.

**What are the implications for public health practice?**

Clinicians should consider arboviral testing for patients with fever or neurologic illness, particularly when mosquitoes and ticks are active. Timely surveillance can be used to identify geographic areas at risk for arboviral infection. Reducing arboviral diseases depends on personal protective measures, vector control, and screening blood and organ donors for WNV.

## Abstract

In the United States, arthropodborne viruses (arboviruses) are primarily transmitted by infected mosquitoes or ticks; rarely, they can be transmitted through blood transfusion or organ transplantation. Whereas a majority of arboviral infections are asymptomatic, symptomatic infections range from mild febrile illness to severe neuroinvasive disease. This report summarizes data for nationally notifiable domestic arboviral diseases with illness onset in 2024. Forty-eight states and the District of Columbia reported 1,955 arboviral disease cases, including 189 (10%) deaths. Although the number of West Nile virus (WNV) disease cases in 2024 was below the 10-year median (2,162), WNV caused most reported arboviral disease cases (1,808; 92%). Powassan virus disease was the second most commonly reported arboviral disease (60; 3%), increasing from a previous high of 49 cases in 2023. A majority of patients with arboviral disease were aged ≥60 years (1,133; 58%), were male (1,223; 63%), and had illness onset during July–September (1,603; 82%). However, La Crosse virus disease occurred almost exclusively among children aged <18 years (35 of 37 cases; median age = 8 years; interquartile interval = 5–10 years). Variations in annual demographic characteristics, disease incidence, distribution, and seasonal temporality of arboviral diseases highlight the importance of quality and ­timely surveillance to increase awareness of disease risk, facilitate testing, and implement control measures. Clinicians should consider arboviral testing for patients with acute febrile or neurologic illness during periods when mosquitoes and ticks are active. Reducing arboviral disease morbidity and mortality relies on use of personal protective measures (e.g., insect repellent and protective clothing), implementing vector-control activities, and screening blood and organ donors for WNV.

## Introduction

Arboviruses are maintained in transmission cycles among arthropods and vertebrate hosts, including humans and other animals (*1*). In the United States, humans typically acquire arboviral infections when they are bitten by an infected mosquito or tick and, although rare, through other routes such as blood transfusion and organ transplantation (*2*). Of the most common nationally notifiable domestic arboviral diseases in the continental United States, five are mosquitoborne (West Nile, La Crosse, Jamestown Canyon, eastern equine encephalitis, and St. Louis encephalitis virus diseases) and one (Powassan virus disease) is tickborne. The majority of arboviral infections are asymptomatic, but disease can range from mild febrile illness to severe, potentially fatal, neuroinvasive disease. Older age, certain chronic medical conditions (e.g., cancer, diabetes, high blood pressure, or kidney disease), and immunosuppression are risk factors for neuroinvasive disease. No prophylactic agents or treatments are available for arboviral diseases; therefore, timely and accurate surveillance is critical to help focus prevention measures (e.g., personal protection against mosquito bites and vector control) to reduce disease morbidity and mortality. This report provides an annual update on domestic arboviral disease case numbers and incidence to increase awareness of the occurrence and characteristics of arboviral diseases in the United States (*3*,*4*).

## Methods

### Data Source

State health departments voluntarily report cases of nationally notifiable domestic arboviral diseases to CDC by location of patient residence through ArboNET, the national arbovirus surveillance system, using standard surveillance case definitions that include clinical and laboratory criteria. Confirmed and probable cases are included in this report; ­cases are further characterized as neuroinvasive (i.e., those with meningitis, encephalitis, acute flac­cid paralysis, or other unspecified neurologic manifestation) or nonneuroinvasive (all other cases).

### Analysis

Confirmed and probable cases with clinically compatible disease and supporting laboratory results were combined in the analysis. The case features of six domestic arboviral diseases (West Nile, Powassan, La Crosse, Jamestown Canyon, eastern equine encephalitis, and St. Louis encephalitis virus diseases) are described and compared with previous years’ data reported to ArboNET to identify trends. Incidence was calculated using U.S. Census Bureau 2024 midyear population estimates as denominators and limited to neuroinvasive disease cases because these cases are more severe and therefore more consistently diagnosed and reported (*1*). A negative binomial regression was chosen for the Powassan case trend analysis to account for overdispersion of the annual count data, with the year variable centered to limit multicollinearity; this analysis was conducted using the SAS proc genmod procedure; all analyses were conducted using SAS (version 9.4; SAS Institute). This activity was reviewed by CDC, deemed not research, and conducted consistent with applicable federal law and CDC policy.*

## Results

### Reported Arboviral Disease Cases

CDC received 1,955 reports of confirmed (594; 30%) and probable (1,361; 70%) arboviral disease cases with illness onset in 2024; 1,485 cases (76%) were neuroinvasive (Table 1). Cases were reported from 647 (21%) of 3,143 U.S. counties and from 48 states and the District of Columbia (DC). WNV accounted for 1,808 (92%) cases, followed by Powassan (60; 3%), La Crosse (37; 2%), Jamestown Canyon (27; 1%), eastern equine encephalitis (19; 1%), St. Louis encephalitis (2; <1%), and unspecified California serogroup viruses^†^ (2; <1%). The specific virus was unknown for two cases with California serogroup virus; these cases were excluded from further description in this report.

**TABLE 1:** Number and percentage of confirmed* and probable^†^ cases of nationally notifiable arboviral disease, by virus type and selected patient characteristics^§^ — United States,^¶^ 2024

Characteristic	Virus type, no. (%)** of cases					
	West Nile	Powassan	La Crosse	Jamestown Canyon	Eastern equine encephalitis	St. Louis encephalitis
**Total, no. (N = 1,953)**	**1,808**	**60**	**37**	**27**	**19**	**2**
**Age group, yrs**						
<18	33 (2)	4 (7)	35 (95)	0 (—)	1 (5)	0 (—)
18–59	720 (40)	10 (17)	1 (3)	7 (26)	7 (37)	2 (100)
≥60	1,055 (58)	46 (77)	1 (3)	20 (74)	11 (58)	0 (—)
**Sex**						
Female	675 (37)	25 (42)	19 (51)	8 (30)	4 (21)	0 (—)
Male	1,133 (63)	35 (58)	18 (49)	19 (70)	15 (79)	2 (100)
**Quarter of illness onset**						
Jan–Mar	3 (<0.01)	4 (7)	0 (—)	0 (—)	0 (—)	0 (—)
Apr–Jun	115 (6)	28 (47)	1 (3)	10 (37)	1 (5)	0 (—)
Jul–Sep	1,523 (84)	14 (23)	32 (86)	13 (48)	18 (95)	1 (50)
Oct–Dec	167 (9)	14 (23)	4 (11)	4 (15)	0 (—)	1 (50)
**Clinical syndrome**						
Nonneuroinvasive	461 (25)	0 (—)	1 (3)	7 (26)	0 (—)	1 (50)
Neuroinvasive^††^	1,347 (75)	60 (100)	36 (97)	20 (74)	19 (100)	1 (50)
Encephalitis	852 (63)	48 (80)	25 (69)	13 (65)	16 (84)	0 (—)
Meningitis	279 (21)	8 (13)	9 (25)	3 (15)	2 (11)	1 (100)
AFP^§§^	55 (4)	4 (7)	0 (—)	0 (—)	0 (—)	0 (—)
Unspecified	161 (12)	0 (—)	2 (6)	4 (20)	1 (5)	0 (—)
**Outcome**						
Hospitalization	1,396 (77)	60 (100)	36 (97)	21 (78)	19 (100)	1 (50)
Death	173 (10)	9 (15)	0 (—)	2 (7)	5 (26)	0 (—)

**Abbreviations:** AFP = acute flaccid paralysis; CSF = cerebrospinal fluid; IgM = immunoglobulin M.

* A confirmed case meets clinical criteria for arboviral disease and at least one of the following laboratory criteria: 1) isolation of virus from, or demonstration of specific viral antigen or nucleic acid in tissue, blood, CSF, or other body fluid; 2) fourfold or higher change in virus-specific quantitative antibody titers in paired sera; 3) virus-specific IgM antibodies in serum with confirmatory virus-specific neutralizing antibodies in the same or a later specimen; or 4) virus-specific IgM antibodies in CSF and a negative test result for other IgM antibodies in CSF for arboviruses endemic in the region where exposure occurred.

^†^ A probable case meets clinical criteria for arboviral infection and virus-specific IgM antibodies in CSF or serum but without other testing.

^§^ Two unspecified California serogroup virus cases were reported but are not included in the table.

^¶^ United States refers to all U.S. states, territories, and the District of Columbia.

** Percentages might not sum to 100 because of rounding.

^††^ Percentages of cases of encephalitis, meningitis, AFP, and unspecified neurologic signs or symptoms are percentages of neuroinvasive cases.

^§§^ Among the 55 West Nile virus disease cases with AFP, 15 (27%) also had encephalitis or meningitis. Among the four Powassan virus disease cases with AFP, all four (100%) also had encephalitis or meningitis.

### WNV Disease

In 2024, a total of 1,808 WNV disease cases were reported; median patient age was 64 years (interquartile interval [IQI] = 51–73 years), and 1,133 (63%) patients were male (Table 1). Three fourths (1,347; 75%) of patients had WNV neuroinvasive disease. A total of 1,396 (77%) patients were hospitalized, including 1,263 (91%) with neuroinvasive disease. Overall, 173 (9.6%) patients with reported WNV disease died, 169 (98%) of whom had neuroinvasive disease (case-fatality rate [CFR] = 13% among patients with neuroinvasive disease). A majority of patients had illness onset during July–September (1,523; 84%). Two neuroinvasive WNV disease deaths were reported in patients who were infected through kidney transplantation from a single deceased organ donor.

WNV disease cases were reported from 591 (19%) of 3,143 counties in 48 states and DC. Texas (363 cases), California (103), and New York (69) accounted for 40% of all U.S. WNV neuroinvasive disease cases. The national incidence of WNV neuroinvasive disease was 0.40 per 100,000 population (Table 2), and incidence was highest in North Dakota (2.76), Nebraska (2.49), and Mississippi (1.43) (Figure). Incidence was higher among males (0.52) than females (0.28) and among adults aged ≥70 years (1.30) than persons in other age groups. Incidence was lowest among children aged <10 years (0.02).

**TABLE 2:** Number and incidence* of confirmed^†^ and probable^§^ cases of nationally notifiable arboviral neuroinvasive disease, by virus type and U.S. Census Bureau division and jurisdiction — United States,^¶^ 2024

U.S. Census Bureau division/Jurisdiction	Virus type, no. (incidence) of cases					
	West Nile	Powassan	La Crosse	Jamestown Canyon	Eastern equine encephalitis	St. Louis encephalitis
**United States**	**1,347 (0.40)**	**60 (0.02)**	**36 (0.01)**	**20 (0.01)**	**19 (0.01)**	**1 (0)**
**New England**	32 (0.21)	28 (0.18)	—**	3 (0.02)	13 (0.08)	—
Connecticut	10 (0.27)	6 (0.16)	—	—	—	—
Maine	1 (0.07)	6 (0.43)	—	—	1 (0.07)	—
Massachusetts	16 (0.22)	11 (0.15)	—	—	4 (0.06)	—
New Hampshire	1 (0.07)	3 (0.21)	—	2 (0.14)	5 (0.35)	—
Rhode Island	3 (0.27)	2 (0.18)	—	1 (0.09)	1 (0.09)	—
Vermont	1 (0.15)	—	—	—	2 (0.31)	—
**Middle Atlantic**	149 (0.35)	6 (0.01)	—	1 (0)	4 (0.01)	—
New Jersey	33 (0.35)	2 (0.02)	—	1 (0.01)	2 (0.02)	—
New York	69 (0.35)	3 (0.02)	—	—	2 (0.01)	—
Pennsylvania	47 (0.36)	1 (0.01)	—	—	—	—
**East North Central**	128 (0.27)	12 (0.03)	4 (0.01)	14 (0.03)	1 (0)	—
Illinois	56 (0.44)	—	—	—	—	—
Indiana	10 (0.14)	—	—	—	—	—
Michigan	24 (0.24)	—	—	8 (0.08)	—	—
Ohio	12 (0.10)	—	4 (0.03)	—	—	—
Wisconsin	26 (0.44)	12 (0.20)	—	6 (0.10)	1 (0.02)	—
**West North Central**	142 (0.65)	14 (0.06)	2 (0.01)	2 (0.01)	—	—
Iowa	16 (0.49)	—	—	—	—	—
Kansas	11 (0.37)	—	—	—	—	—
Minnesota	26 (0.45)	14 (0.24)	1 (0.02)	2 (0.03)	—	—
Missouri	12 (0.19)	—	1 (0.02)	—	—	—
Nebraska	50 (2.49)	—	—	—	—	—
North Dakota	22 (2.76)	—	—	—	—	—
South Dakota	5 (0.54)	—	—	—	—	—
**South Atlantic**	130 (0.19)	—	17 (0.02)	—	1 (0)	—
Delaware	1 (0.10)	—	—	—	—	—
District of Columbia	2 (0.28)	—	—	—	—	—
Florida	15 (0.06)	—	—	—	—	—
Georgia	39 (0.35)	—	—	—	—	—
Maryland	24 (0.38)	—	—	—	—	—
North Carolina	27 (0.24)	—	15 (0.14)	—	1 (0.01)	—
South Carolina	15 (0.27)	—	1 (0.02)	—	—	—
Virginia	5 (0.06)	—	—	—	—	—
West Virginia	2 (0.11)	—	1 (0.06)	—	—	—
**East South Central**	87 (0.44)	—	12 (0.06)	—	—	—
Alabama	27 (0.52)	—	—	—	—	—
Kentucky	8 (0.17)	—	—	—	—	—
Mississippi	42 (1.43)	—	—	—	—	—
Tennessee	10 (0.14)	—	12 (0.17)	—	—	—
**West South Central**	450 (1.04)	—	1 (0)	—	—	1 (0)
Arkansas	12 (0.39)	—	—	—	—	—
Louisiana	38 (0.83)	—	—	—	—	—
Oklahoma	37 (0.90)	—	—	—	—	—
Texas	363 (1.16)	—	1 (0)	—	—	1 (0)
**Mountain**	124 (0.47)	—	—	—	—	—
Arizona	23 (0.30)	—	—	—	—	—
Colorado	40 (0.67)	—	—	—	—	—
Idaho	4 (0.20)	—	—	—	—	—
Montana	3 (0.26)	—	—	—	—	—
Nevada	15 (0.46)	—	—	—	—	—
New Mexico	24 (1.13)	—	—	—	—	—
Utah	14 (0.40)	—	—	—	—	—
Wyoming	1 (0.17)	—	—	—	—	—
**Pacific**	105 (0.19)	—	—	—	—	—
Alaska	—	—	—	—	—	—
California	103 (0.26)	—	—	—	—	—
Hawaii	1 (0.07)	—	—	—	—	—
Oregon	—	—	—	—	—	—
Washington	1 (0.01)	—	—	—	—	—

**Abbreviations:** CSF = cerebrospinal fluid; IgM = immunoglobulin M.

* Cases per 100,000 population, based on July 1, 2024, U.S. Census Bureau population estimates.

^†^ A confirmed case meets clinical criteria for arboviral disease and at least one of the following laboratory criteria: 1) isolation of virus from, or demonstration of specific viral antigen or nucleic acid in tissue, blood, CSF, or other body fluid; 2) fourfold or higher change in virus-specific quantitative antibody titers in paired sera; 3) virus-specific IgM antibodies in serum with confirmatory virus-specific neutralizing antibodies in the same or a later specimen; or 4) virus-specific IgM antibodies in CSF and a negative test result for other IgM antibodies in CSF for arboviruses endemic in the region where exposure occurred.

^§^ A probable case meets clinical criteria for arboviral infection and virus-specific IgM antibodies in CSF or serum but without other testing.

^¶^ United States refers to all U.S. states, territories, and the District of Columbia.

** Dashes indicate no reported cases.

**FIGURE:**
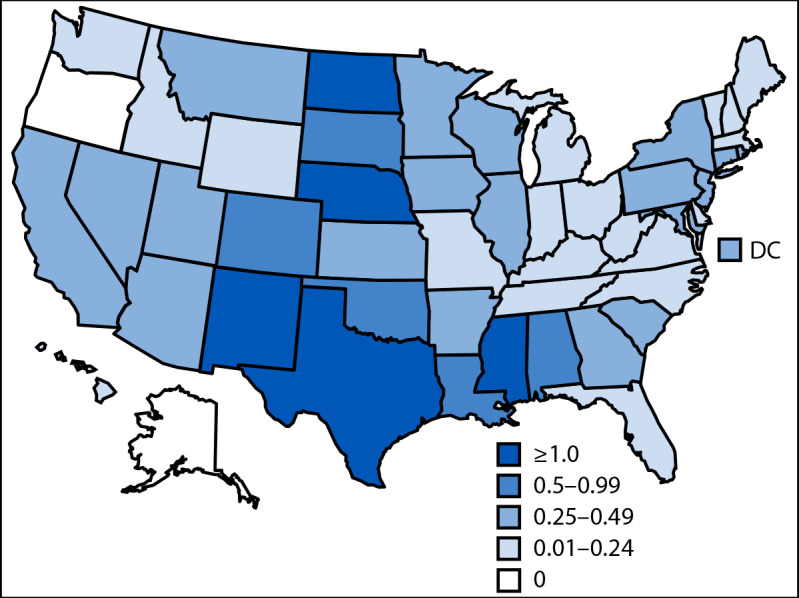
Incidence* of confirmed^†^ and probable^§^ cases of neuroinvasive West Nile virus disease, by state — United States,^¶^ 2024 **Abbreviations:** CSF = cerebrospinal fluid; DC = District of Columbia; IgM = immunoglobulin M. * Cases per 100,000 population, based on July 1, 2024, U.S. Census Bureau population estimates. ^†^ A confirmed case meets clinical criteria for arboviral disease and at least one of the following laboratory criteria: 1) isolation of virus from, or demonstration of, specific viral antigen or nucleic acid in tissue, blood, CSF, or other body fluid; 2) fourfold or higher change in virus-specific quantitative antibody titers in paired sera; 3) virus-specific IgM antibodies in serum with confirmatory virus-specific neutralizing antibodies in the same or a later specimen; or 4) virus-specific IgM antibodies in CSF and a negative test result for other IgM antibodies in CSF for arboviruses endemic in the region where exposure occurred. ^§^ A probable case meets clinical criteria for arboviral infection and virus-specific IgM antibodies in CSF or serum but without other testing. ^¶^ United States refers to all U.S. states, territories, and DC.

The 1,808 reported WNV disease case count in 2024 is below the 10-year median of 2,162 cases (IQI = 1,132–2,628). The 456 cases reported in Texas are more than double the number reported in Texas in 2023 (163).

### Powassan Virus Disease

In 2024, a total of 60 Powassan virus disease cases were reported. The median patient age was 69 years (IQI = 60–75 years), with four patients (7%) aged <18 years, and 35 (58%) patients were male (Table 1). All patients were hospitalized with neuroinvasive disease, and nine died (CFR = 15%). Cases occurred throughout the year, and approximately one half (28; 47%) had illness onset during April–June.

Powassan virus disease cases were reported from 45 counties across 10 states. Nationally, the incidence of neuroinvasive Powassan virus disease was 0.02 per 100,000 population, with the highest incidence in Maine (0.43), Minnesota (0.24), New Hampshire (0.21), and Wisconsin (0.20) (Table 2). From 2023 to 2024, Minnesota and Wisconsin experienced a combined 160% relative increase in cases, from 10 to 26. Nationally, the number of cases reported in 2024 was above the 10-year median of 23 cases (IQI = 21–43), and during the previous 21 years, cases increased with an annual growth rate of 17.5% (95% CI = 13.6–21.6; negative binomial regression; p<0.001).

### La Crosse Virus Disease

In 2024, a total of 37 La Crosse virus disease cases were reported; 35 (95%) occurred among children aged <18 years (median age = 8 years; IQI = 5–10 years), and 19 (51%) occurred among females. Overall, 36 (97%) patients were hospitalized, 36 (97%) had neuroinvasive disease, and none died (Table 1). Illness onset occurred during July–September among 32 (86%) patients. Cases were reported from 24 counties in eight states. Overall, 75% of neuroinvasive La Crosse virus disease cases were reported from North Carolina (15) and Tennessee (12), representing a relative increase of 170% from five cases in each state reported in 2023, although the number of cases in 2024 was below the 10-year median (55 cases; IQI = 35–80). The national incidence of neuroinvasive La Crosse virus disease was 0.01 per 100,000 population (0.05 among children aged <18 years) and was highest in Tennessee (0.17), North Carolina (0.14), and West Virginia (0.06) (Table 2).

### Jamestown Canyon Virus Disease

In 2024, a total of 27 cases of Jamestown Canyon virus disease was reported; the median patient age was 66 years (IQI = 53–76 years), and 19 (70%) patients were male. Twenty-one (78%) patients were hospitalized, 20 (74%) had neuroinvasive disease, and two (7%) died (CFR = 10% among patients with neuroinvasive disease) (Table 1). Illness onset occurred during July–September for 13 (48%) patients and April–June for 10 (37%). Cases were reported from 23 counties in six states. The highest numbers of cases were reported from Wisconsin (10), Michigan (eight), and Minnesota (four). The national incidence of neuroinvasive Jamestown Canyon virus disease was 0.01 per 100,000 population, with the highest incidences in New Hampshire (0.14), Wisconsin (0.10), Rhode Island (0.09), and Michigan (0.08) (Table 2). The number of Jamestown Canyon virus disease cases in 2024 was similar to the 10-year median (21 cases; IQI = 12–41).

### Eastern Equine Encephalitis Virus Disease

In 2024, a total of 19 cases of eastern equine encephalitis virus disease were reported; the median patient age was 68 years (IQI = 53–78 years), and 15 (79%) patients were male. All patients were hospitalized with neuroinvasive disease, and five died (CFR = 26%) (Table 1). Eighteen (95%) patients had illness onset during July–September. Cases were reported from 12 counties in nine states. The highest numbers of cases were reported by New Hampshire (five) and Massachusetts (four). The national incidence of neuroinvasive eastern equine encephalitis virus disease was 0.01 per 100,000, and rates were highest in New Hampshire (0.35) and Vermont (0.31) (Table 2). The number of reported eastern equine encephalitis disease cases in 2024 was the highest since 2019 and higher than the 10-year median of 6.5 (IQI = 5–8).

### St. Louis Encephalitis Virus Disease

Two cases of St. Louis encephalitis virus disease were reported in 2024, both in men aged <60 years. One patient was hospitalized with neuroinvasive disease, and both survived (Table 1). Illness onsets occurred in August and November. One neuroinvasive case was reported from Texas corresponding to an incidence of <0.01 per 100,000 population (Table 2). The number of St. Louis encephalitis virus disease cases was the lowest since 2013 and substantially lower than the 10-year median of 16.5 cases (IQI = 10–21).

## Discussion

Domestic arboviruses occur in arthropod and animal hosts throughout the continental United States. Local ecology and climate, especially temperature and moisture, host immunity, and human activities all influence disease occurrence. In 2024, WNV was the most common domestic arbovirus, but the number of reported cases was below the 10-year median. High case counts are often driven by regional outbreaks, as in 2024 when 456 WNV disease cases reported from Texas accounted for 25% of all WNV cases, highlighting geographic heterogeneity in transmission. The numbers of eastern equine encephalitis and Powassan virus disease cases in 2024 were higher than the previous 10-year medians, which is especially concerning because these two arboviral diseases are associated with the highest CFRs among domestic arboviral diseases (26% and 15%, respectively). Eastern equine encephalitis is characterized by periodic surges over time and in certain geographic areas; the number of cases reported in 2024 (19) was the third highest reported since 2003, following 2019 (38) and 2005 (21) (*5*–*7*). In contrast, the number of Powassan virus disease cases has been increasing by an average of 17.5% annually during the past 21 years. The reasons for this might be related to an actual increase in incidence, improved health care provider awareness, expanded availability of diagnostic testing at commercial and state public health laboratories, or potential expansion of the vector range (*8*).

In the United States, although adults aged ≥60 years are at higher risk for most neuroinvasive arboviral disease and poor outcomes compared with younger persons, who are more likely to have an asymptomatic or mild, febrile illness (*1**,**9*), La Crosse virus disease occurs more commonly among children. In 2024, La Crosse virus disease accounted for 54% of the 63 neuroinvasive arboviral disease cases reported among children aged <18 years, followed by WNV disease (35%) and Powassan virus disease (6%). Arboviral morbidity among children can be severe, including neuroinvasive disease and death (*10*).

One WNV transplant cluster associated with two neuroinvasive disease deaths was identified in 2024. Although rare, arboviral transmission through solid organ transplantation is often associated with high morbidity and mortality among organ recipients, likely related to their immunosuppression (*2*). Organ donors in the United States, unlike blood donors, were not universally screened for WNV. However, a policy that requires seasonal WNV screening (July 1–October 31) for all potential living and deceased solid organ donors was implemented in June 2026.

### Limitations

The findings in this report are subject to at least three limitations. First, cases can be misclassified because inclusion in ArboNET does not require clinical signs and symptoms or diagnostic laboratory test results. Second, ArboNET is a passive surveillance system that relies on patients seeking health care, appropriate testing by clinicians, and reporting of arboviral disease cases; thus, prevalence is likely underestimated. Conservative estimates based on historical ratios and assuming comparable national reporting patterns, population characteristics, and environmental conditions demonstrated that 30–70 nonneuroinvasive disease cases occur for every reported case of WNV neuroinvasive disease (*9*); accordingly, 40,410–94,290 nonneuroinvasive WNV disease cases likely occurred nationally in 2024, although only 461 (0.5%–1% of the estimated total) were reported. Finally, the average annual growth rate estimated for the Powassan virus disease trend is limited to the 21-year historical period and is not necessarily a predictor of future trends.

### Implications for Public Health Practice

High-quality and timely surveillance is critical to understanding the epidemiology, seasonality, and geographic distribution of domestic arboviruses, which can facilitate clinical recognition, identification of outbreaks, messaging activities to the public and health care providers, and vector-control activities. WNV and other arboviral disease testing should be considered by clinicians for patients with acute febrile or neurologic illness, including recipients of organ transplants or blood transfusions, particularly during times when mosquitoes and ticks are active. Health care providers who order testing for a specific causative agent should take into consideration the timing of illness onset, type and location of exposures, patient age, and underlying health status.

The seasonality of arboviral diseases varies according to the vectors responsible for transmitting them; ticks, which transmit Powassan virus, are active earlier and later during the season compared with mosquitoes that typically have peak activity during July–September. Therefore messaging (e.g., media, newsletters, and education at trailheads or parks) to increase public and health care provider awareness can be tailored based on locally circulating arboviruses and the activity of their vectors.

Approximately 25 years after the detection of WNV in the United States, effective tools for diagnosis, prevention, and treatment are lacking. Consistently high WNV disease incidence each year indicates that further research and development of additional strategies, such as human vaccines and prophylactic or therapeutic monoclonal antibodies for persons at high risk for severe illness, are needed. Development of rapid diagnostic tests can facilitate earlier and more reliable diagnosis, which would also improve patient care, disease surveillance, timely public health messaging, and the ability to conduct clinical trials.

Because no specific treatments (e.g., antiviral medications) are available for domestic arboviral diseases, clinical management is supportive. Likewise, no prophylactic agents (e.g., human vaccines) are available to prevent domestic arboviral diseases; therefore, prevention and control rely on use of personal protective measures (e.g., using insect repellent registered by the Environmental Protection Agency and wearing protective clothing), vector-control activities both at household and community levels (CDC | Ticks | Preventing Tick Bites and CDC | Mosquitoes | Mosquito Control), and WNV blood and organ donor screening to minimize transfusion- and transplant-associated transmission.

## Footnotes

* 45 C.F.R. part 46, 21 C.F.R. part 56; 42 U.S.C. Sect. 241(d); 5 U.S.C. Sect. 552a; 44 U.S.C. Sect. 3501 et seq.

† Unspecified California serogroup virus reports belong to the California serogroup (e.g., La Crosse, Jamestown Canyon, snowshoe hare, and California encephalitis viruses), but the specific virus is unknown or not identified.
